# Evaluation of Bait Station Density for Oral Rabies Vaccination of Raccoons in Urban and Rural Habitats in Florida

**DOI:** 10.3390/tropicalmed2030041

**Published:** 2017-08-22

**Authors:** Betsy S. Haley, Timothy P. Algeo, Brian Bjorklund, Anthony G. Duffiney, Robert Edwin Hartin, Ashlee Martin, Kathleen M. Nelson, Richard B. Chipman, Dennis Slate

**Affiliations:** 1 United States Department of Agriculture, Animal and Plant Health Inspection Service, Wildlife Services, National Rabies Management Program, 59 Chenell Dr., Suite 2, Concord, NH 03301, USA; betsy.s.haley@aphis.usda.gov (B.S.H.); kathleen.m.nelson@aphis.usda.gov (K.M.N.); richard.b.chipman@aphis.usda.gov (R.B.C.); dennis.slate@aphis.usda.gov (D.S.); 2 United States Department of Agriculture, Animal and Plant Health Inspection Service, Wildlife Services, 59 Chenell Dr., Suite 7, Concord, NH 03301, USA; timothy.p.algeo@aphis.usda.gov; 3 United States Department of Agriculture, Animal and Plant Health Inspection Service, Wildlife Services, 9 Main St., Suite 1M, Sutton, MA 01590, USA; brian.bjorklund@aphis.usda.gov; 4 United States Department of Agriculture, Animal and Plant Health Inspection Service, Wildlife Services, 2803 Jolly Rd., Suite 100, Okemos, MI 48864, USA; anthony.g.duffiney@aphis.usda.gov; 5 United States Department of Agriculture, Animal and Plant Health Inspection Service, Wildlife Services, 2820 E University Ave., Gainesville, FL 32641, USA; edwin.hartin@aphis.usda.gov

**Keywords:** bait stations, raccoons, rabies, vaccine, oral rabies vaccination

## Abstract

Efforts to eliminate the raccoon variant of the rabies virus (raccoon rabies) in the eastern United States by USDA, APHIS, Wildlife Services and cooperators have included the distribution of oral rabies vaccine baits from polyvinyl chloride (PVC) bait stations in west-central Florida from 2009 to 2015. Achieving sufficient vaccine bait uptake among urban raccoons is problematic, given limitations on aerial and vehicle-based bait distribution for safety and other reasons. One or three bait stations/km^2^ were deployed across four 9-km^2^ sites within rural and urban sites in Pasco and Pinellas Counties, Florida. Based on tetracycline biomarker analysis, bait uptake was only significantly different among the urban (Pinellas County) high and low bait station densities in 2012 (*p* = 0.0133). Significant differences in RVNA were found between the two bait station densities for both urban 2011 and 2012 samples (*p* = 0.0054 and *p* = 0.0031). Landscape differences in terms of urban structure and human population density may modify raccoon travel routes and behavior enough for these differences to emerge in highly urbanized Pinellas County, but not in rural Pasco County. The results suggest that, in urban settings, bait stations deployed at densities of >1/km^2^ are likely to achieve higher seroprevalence as an index of population immunity critical to successful raccoon rabies control.

## 1. Introduction

Globally, rabies kills approximately 59,000 humans annually, and impacts on human and animal health result in a significant economic burden [1]. In the United States, the cost of living with the virus ranges from $245–510 million annually [2]. Oral rabies vaccination (ORV) is an effective and socially-acceptable approach to wildlife rabies control [3]. ORV has been used to control fox rabies in western Europe [4,5] and in Canada [6,7,8]. In the U.S., ORV is currently aimed at the elimination and prevention of new epizootics of canine rabies in coyotes (*Canis latrans*) [9,10], the elimination of rabies in gray fox (*Urocyon cinereoargenteus*) in Texas [11] and the containment and elimination of the raccoon (*Procyon lotor*) variant of the rabies virus (raccoon rabies) in the eastern U.S. [3].

While there are many variants of the rabies virus, and many vector species, raccoon rabies is primarily perpetuated within the raccoon. Raccoons often occur at extremely high population densities along the rural-urban interface, and are ecological generalists [12]. Raccoon rabies has spread rapidly in the abundant raccoon populations of eastern North America; however, the virus has not moved west of the Appalachian Mountain Range. Using this range as a natural barrier, USDA, APHIS, Wildlife Services (WS), National Rabies Management Program (NRMP) has implemented a large-scale ORV program to prevent the westward spread of raccoon rabies [3]. WS NRMP is conducting cooperative ORV operations to continue preventing the spread of raccoon rabies into the mid-western U.S. and eastern Canada (Phase I), and has begun work towards its elimination from the eastern U.S. (Phase II) [3], much of which is highly urbanized.

Bait stations for distribution of oral rabies vaccine baits have become an increasingly important bait delivery method in urban areas where aerial and vehicle-based (or hand) vaccine bait delivery is hampered by high human and pet densities, and in rural areas where raccoon densities are low, but target species may be concentrated in smaller localized populations, reducing the need to widely broadcast vaccine baits. Bait station use began in New York in 2003, and in key locations in Massachusetts in 2006 [13,14], with important questions regarding optimal design and effectiveness left unanswered. Although bait station design and deployment has been evaluated, including modification to reduce non-target uptake, especially by opossums (*Didelphis virginiana*), future design improvements and optimized strategies for their use require additional study [13,15,16]. Opossums are a non-target species due to their low incidence of rabies. They are attracted to vaccine baits and are able to remove baits from the bait stations with little difficulty. Direct competition with raccoons for the baits can confound rabies management efforts [17,18].

To better understand the best management strategies for using bait stations to control raccoon rabies in central Florida during 2011 and 2012, presence of tetracycline (TTCC) biomarker and rabies virus neutralizing antibodies (RVNA) as indices of bait station performance [3] were compared between two bait station densities in rural and urban settings using fishmeal polymer (FMP) baits containing RABORAL V-RG^®^ (Merial, Athens, GA, USA) vaccine. It was predicted that placing 3 bait stations/km^2^ would result in significantly higher RVNA and TTCC percentages than having 1 bait station/km^2^ among the urban study sites, and that there would be no significant difference between 3 bait stations/km^2^ and 1 bait station/km^2^ among the rural study sites.

## 2. Materials and Methods 

**Figure 1:**
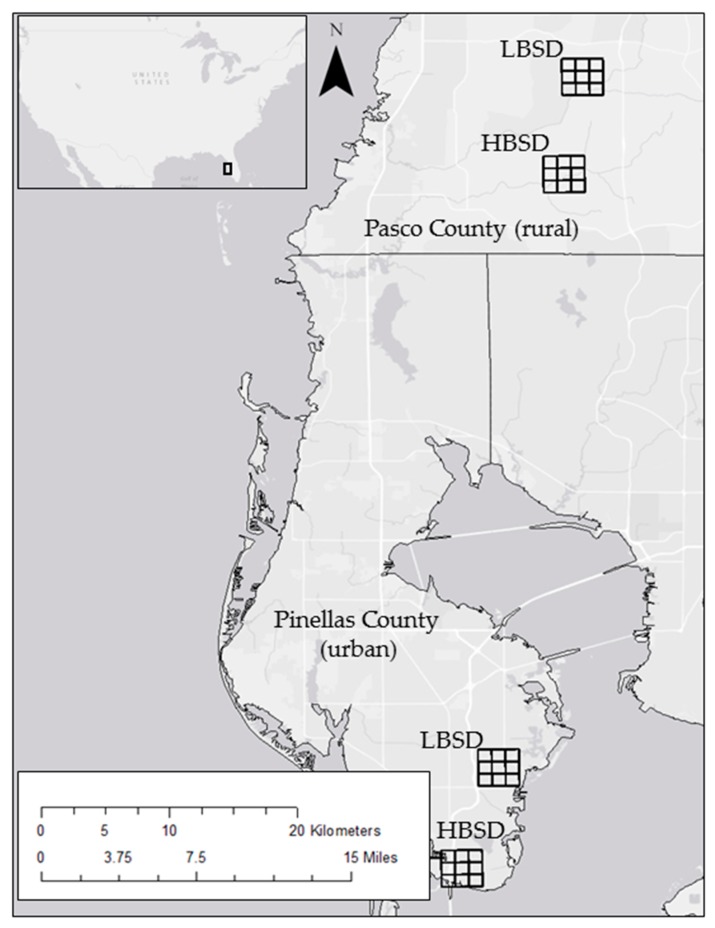
Location of 2011 and 2012 bait station sites within rural (Pasco County) and urban (Pinellas County) locations. The LBSD sites in each county had one bait station/km^2^, while the HBSD sites in each county had three bait stations/km^2^.

Rural study sites were selected within the Starkey Wilderness Preserve in Pasco County, Florida, which is owned and managed by the Southwest Florida Water Management District (SWFWMD), and urban sites were selected within St. Petersburg in Pinellas County, Florida (Figure 1). The rural study sites were dominated by oak (*Quercus* spp.) and pine (*Pinus* spp.) woodlands, with few to no houses in the area. In this study, there were 87 houses within the northeast corner of the study site and bait stations were set at least 0.04 km from the property lines. The rural study sites were interspersed with dirt trails maintained by SWFWMD. An understory of scrub and shrub species was throughout the rural study sites. The urban sites were located within St. Petersburg, Florida, which had a population of approximately 245,300 at the time of the studies, with a population density of approximately 3970 people/mi^2^ (or 1533 people/km^2^) [19]. The landscape was dominated by residential and commercial properties. The study sites will be referenced as rural (Pasco County) or urban (Pinellas County) high bait station density (HBSD)—those sites with 3 bait stations/km^2^, and rural or urban low bait station density (LBSD)—those sites with 1 bait station/km^2^. 

**Figure 2:**
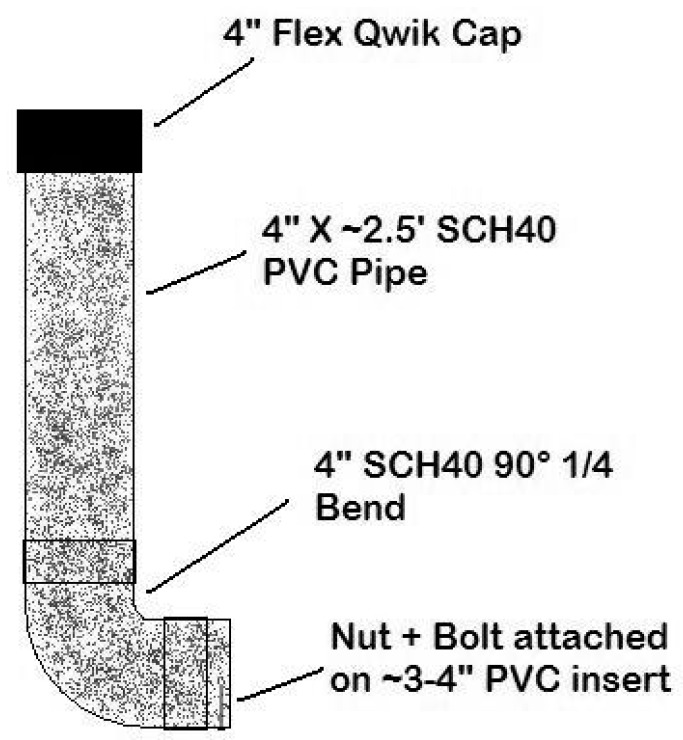
Bait station specifications used in Pasco and Pinellas Counties, Florida, during 2011–2012.

Bait stations were constructed of 2.5-foot sections of 4-inch diameter polyvinyl chloride (PVC) schedule 40 pipe, painted in camouflaged colors to reduce the likelihood of human tampering. Open PVC-tops were covered with 4-inch flexible Qwik^®^ (United States Plastic Corp, Lima, OH, USA) caps to prevent rain and bait access for animals from the top. PVC elbows (90 degree angle) were attached to the 2.5-foot PVC section bottoms, and a 3–4 inch PVC pipe extended from the elbow with a nut and bolt to prevent baits from falling out of the bait station (Figure 2). The bolt acts as a stop to prevent the baits from sliding out and the nut holds the bolt in place. This design was based on the bait station design by Boulanger et al. [13], and then modified to accommodate more baits at one time.

Bait stations were deployed over 10 consecutive nights during 9–20 May 2011 and 21 February–5 March 2012. Due to the number of bait stations to be deployed, not all bait stations were set on the same day. Each bait station area was active for 10 days, though the total number of study days was >10 days. Study sites were selected within 5 km of previous WS raccoon density study or bait station study sites to provide working knowledge of the raccoon populations in the study areas. The raccoon density in Pasco County was estimated at approximately 10 raccoons/km^2^ during a density study conducted in 2011; however, there were no density studies conducted in Pinellas County. Target bait densities on all sites were 75/km^2^, the standard base rate for distributing baits based on current raccoon densities [3]. The bait densities were kept constant across the study sites to ensure study sites could be compared to one another. Two 9-km^2^ study sites were selected (within one habitat type (i.e., woodland-dominated or urban/residential-dominated) to the greatest degree possible) at least 5 km apart in each of the two counties (urban LBSD and HBSD, and rural LBSD and HBSD). Thirty-six bait stations were deployed in each county, and 1350 vaccine baits containing TTCC hydrochloride as a biomarker were deployed on Day 0 in each county. The FMP baits containing RABORAL V-RG^®^ vaccine were 1.25 inch × 1.25 inch × 0.5 inch brown square blocks made of fishmeal. Inside the bait was a sachet sealed in the block with wax. The pink liquid inside the sachet was the vaccine. The amount of vaccine was intended to be a single dose.

The LBSD site in each county was equipped with one bait station/km^2^, containing 75 baits each (9 bait stations/site). The HBSD site in each county was equipped with three bait stations/km^2^ containing 25 baits each (27 bait stations/site). Even distribution of the bait stations within the rural sites was possible due to the rural nature (woodlands with scrub/shrub understory) of the site, and single ownership; only vegetation and a lack of trails influenced bait station distribution. These sites were dominated by saw palmettos (*Serenoa repens*), oaks, and pines. Thorny vines, like greenbrier (*Smilax bona-nox*), made human movement difficult. Access to trails in 2012 that were available for use in 2011 was reduced by storm damage. The distribution of bait stations within the urban HBSD site was clustered in several 1-km^2^ sections based on landowner permission, vegetative cover and the need to hide bait stations from the view of the public to reduce tampering. 

Bait stations were visited three to five times during each study period to monitor activity, equipment and site conditions. In the urban sites, four infrared automated cameras were positioned in LBSD, and six in HBSD, while in the rural sites, five were set in LBSD, and six in HBSD. All cameras were Moultrie^®^ (EBSCO Industries, Inc., Birmingham, AL, USA), and both Gamespy D55IR and I40 Digital Game Camera models were used. The D55IR was a 5.0-megapixel camera and accepted SD cards of up to 16 GB. The I40 was a 4.0-megapixel camera and accepted SD cards of up to 4 GB. Photos were set at high image quality, with 1-minute activation intervals and with the multi-shot function turned on to capture 3 photos for each activation on both cameras. No flash was used; only the infrared flash was used at night. Sensor, aperture, and focal lengths were adjusted automatically as needed; these were not changed from the original setting as there was no means to adjust them. Cameras were set 12–24 inches from the ground, a minimum of 3 feet from the bait station, and aimed toward bait station openings to determine the species (raccoon vs. non-target) taking bait. Direction of the cameras was not accounted for, as most of the bait stations were set within clumps of vegetation so direct sunlight was not a factor. Each camera was given a unique ID number, which was printed on the photos to enable proper location of the photos. While setting the bait stations and cameras, the bait station number was recorded along with the corresponding camera ID. Any removal of vegetation in the rural sites that may have interfered with the cameras capturing photos was kept to a minimum so as not to make changes to the habitat that could deter animal visitations. In the urban sites, no vegetation changes were made, since the bait stations were set primarily on private property, and damage the landowners’ plants was not desired. Camera event counters were reset during each site visit, and the time between photographs was minimized. During each bait station visit, the following information was recorded: date of visit, bait station ID, camera type, number of photos on camera (since last visit), number of images by species, and bait condition. The photos were viewed on a laptop computer. Each new event was determined by a 15-minute interval between photos showing individual animals. If the animal could be accurately identified by its markings as the same animal in the previous event photo (15 min prior), then this was considered a new event but not a new individual. If a bait station was emptied prior to the end of the of the 10-day study period, it was removed along with the camera, if one was associated with the bait station, to reduce tampering and damage to the bait station.

Trapping began 24 days after bait stations were removed to allow sufficient time for TTCC biomarker deposition and RVNA development, and to approximate the time between standard ORV bait distribution and post-ORV sampling. Trapping occurred within 0.5 km of each study site to optimize capture rates, and ≥30 unique raccoons/study site were targeted to facilitate TTCC biomarker and serological analyses. Trapping was completed 84 days post-station removal. Raccoons were marked with a metal #4 ear tag (National Band & Tag Co., Newport, KY, USA) stamped with a unique identifying number so as to identify each individual raccoon captured. Given that all past and present captured raccoons are marked in the same fashion, any animals recaptured from previous studies could be easily identified and removed from testing if treated in a manner (i.e., given vaccination by injection) that would affect this study’s results. Standard biological samples were collected, including blood sera to determine vaccine-induced immunity, and first premolar (PM1) teeth for biomarker evaluation. Although biomarking frequently occurs in fewer animals than actually demonstrate vaccine-induced serological responses due to extraction of a first premolar from live tapped and released raccoons, it remains useful when considered with other vaccination assessment tools [20,21]. The teeth were labeled and prepared for shipment to Matson’s Laboratory, LLC (Manhattan, MT, USA) where the tetracycline biomarker analysis was performed. Methods used for this test were performed as stated in Algeo et al. [20] and Linhart and Kenelly [22]. Rabies virus neutralizing antibody tests were conducted at the Centers for Diseases Control and Prevention (CDC) in Atlanta, GA, using the rapid fluorescent focus inhibition test (RFFIT). Methods for this test were performed as stated in Smith et al. [23] and CDC [24]. A cut-off of both ≥0.05 and ≥0.1 IU/mL were used to indicate a positive RVNA response. It was desired to determine if there was a detectable difference with using the lower 0.05 IU/mL versus the higher 0.1 IU/mL.

Fisher’s exact tests were used to compare RVNA rates within and between treatments and sites. GraphPad QuickCalcs (GraphPad Software, Inc., La Jolla, CA, USA) statistical software was used for analyses [25], with α = 0.05.

## 3. Results

**Figure 3:**
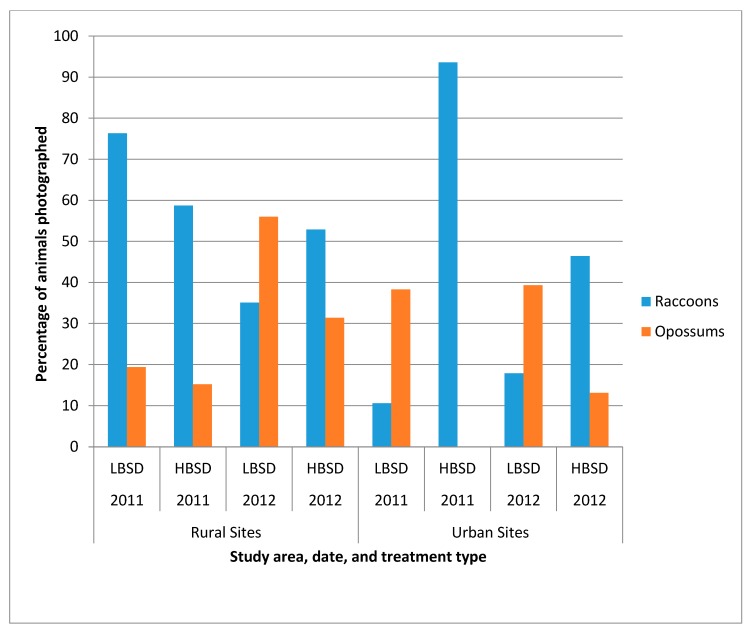
Comparison of raccoon and non-target opossum ratios in automatic camera images at LBSD and HBSD sites in rural and urban environments in Pasco and Pinellas Counties, Florida, during 2011–2012.

The photographs captured by each camera were examined and the number of individual animals photographed was documented. One camera was removed from the rural HBSD counts in both 2011 and 2012 for lack of photographs showing any individual animals. In 2011, total camera days were 44 (rural LBSD, 5 cameras), 42 (rural HBSD, 5 cameras), 40 (urban LBSD, 4 cameras) and 22 (urban HBSD, 6 cameras). Total camera days in 2012 were 34 (rural LBSD, 5 cameras), 24 (rural HBSD, 5 cameras), 40 (urban LBSD, 4 cameras) and 31 (urban HBSD, 6 cameras). Photographs were analyzed for individual identifiable animals by markings. If an animal was not identified as the same with certainty, then it was counted as a new individual. Raccoons were photographed more frequently in five of the eight sampling periods than were opossums, the primary non-target species in the area. However, during 2012 in the rural LBSD site, and during 2011 and 2012 in the urban LBSD site, more opossums were photographed (Figure 3). Only in the rural LBSD site did raccoons predominate amongst the photographed individuals in 2011, and opossums predominated in 2012.

A total of 244 raccoons was trapped and sampled during 2011 and 2012; seven of these were removed from the results due to previous vaccination by injection during 2011. RVNA rates ranged from 6.3% (urban LBSD 2012) to 53.8% (rural HBSD 2012) (Table 1). The HBSD sites resulted in more elevated RVNA rates in 2012 (53.8% and 51.6%) than did the LBSD sites (44.4% and 6.3%). The 2012 rural and urban HBSD sites also had more elevated RVNA rates (53.8% and 51.6%) than both rural and urban HBSD in 2011 (35.1% and 45.2%). Tetracycline biomarker was present in more teeth collected from both rural and urban HBSD sites in 2012 (30.4% and 33.3%) than in both 2012 rural and urban LBSD sites (26.9% and 0.0%) (Table 1). 

Bait removal from bait stations varied between sites and between years. The nine urban LBSD bait stations started each year with a total of 675 baits, and had only 179 baits removed (26.5%) by the end of the 10-day study period in 2011 and 265 baits removed (39.3%) in 2012 (Table 1). A greater percentage of baits were taken from bait stations in 2012 than 2011. Within the urban sites, a larger number of baits were taken from the bait stations in the HBSD site regardless of year. However, within the rural areas, one bait station in the HBSD site did not have any baits removed in 2012 while all the baits within the LBSD site were removed from the bait stations (Table 1).

RVNA rates were significantly higher (*p* = 0.0054 and 0.0031, respectively) in urban HBSD sites in 2011 and 2012 using an RVNA cutoff of ≥0.05 IU/mL, indicating a relationship with increased bait station density in urban areas. However, the rural sites did not differ (Table 2A). RVNA rates were significantly higher (*p* = 0.0081 and 0.0031, respectively) in urban HBSD sites in 2011 and 2012 using an RVNA cutoff of ≥0.1 IU/mL, indicating a relationship with increased bait station density in urban areas. However, the rural sites did not differ (Table 2B).

Tetracycline biomarker was higher in the urban HBSD site in 2012 (*p* = 0.0133; Table 3). No significant differences were found between the rural sites or the 2011 urban sites.

## 4. Discussion

Achieving sufficient vaccine bait uptake among urban raccoons is critical. Limitations on aerial and vehicle-based (hand) bait distribution for safety and other reasons necessitate finding other bait distribution means and optimized strategies for achieving RVNA rabies management goals. Bait stations represent one potential tool for specific settings that may achieve management goals while reducing non-target bait loss and pet and human bait contact, mitigating many concerns from managers, cooperating agencies, and the public. Even though the bait stations were studied in May 2011 and February 2012, it was anticipated that these differences in times of year did not have any impact on the results. Though warmer temperatures may impact raccoon movements during the day, when looking at the total baits removed per year, it does not appear that time of year had any effect on the results of this study. It was believed that raccoon movement was sufficient to ensure that many raccoons came in contact with the baits in both years. The warmer temperatures during the 2011 trapping period may have been thought to negatively impact the capture rate, but capturing raccoons within the urban LBSD site in 2012 proved to be more difficult (*n* = 16; Table 1).

Bait removal from bait stations resulted in higher RVNA percentages in the urban HBSD sites, irrespective of RVNA cutoff level. The lack of uptake in the urban LBSD site (<40% of baits removed in both 2011 and 2012 out of 675 baits) may be due to a perceived relatively low localized raccoon density at the time of this study, which can be evidenced from the relatively low percentage of raccoon photos in this area in 2011 and 2012 (Figure 3). A lack of travel corridors due to roads through the area, fenced and relatively barren yards, few park and recreational areas, and people and pet interference may also have had negative impacts on raccoon movements through this site. In contrast, the urban HBSD site (74.2% and 94.7% of baits removed in 2011 and 2012, respectively, out of 675 baits) contained multiple parks, ideal tree cover, food resources, and habitats for raccoons, as well as a golf course and conservation areas with multiple fresh water ponds. Many of the house lots in this site contained several large trees as potential denning sites.

Domestic dogs (*Canis familiaris*) and cats (*Felis catus*) were both captured in photos only at the urban bait stations; however, none were documented taking a bait from the bait stations. Therefore they were not reported in the results. Since these animals did not take any baits and were not observed eating a bait that was on the ground, neither dogs nor cats were considered a non-target species for the bait.

Greater bait removal from the bait stations in 2012 than in 2011 may be due to identical bait station locations in both years, possibly resulting in bait stations being more easily found in the second year. Several authors [26,27,28,29,30] have documented learned behavior in raccoons—from traversing a maze after being shown the end, to pulling a lever for a reward after watching someone pull the same lever, to gaining access to garbage cans after lid modifications have been made. Female raccoons with young were documented in the 2011 photos. It is possible that those young returned the next year after learning the bait stations provided bait. A single bait station in the rural HBSD site had no baits taken, possibly due to the presence of acrobat ants (*Crematogaster ashmeadi*) that were observed covering the baits. These native fire ants potentially reduced the bait scent attractant to wildlife. Fire ants have been observed by multiple wildlife personnel throughout the southeastern U.S. covering bait in traps, as well as vaccine baits on the ground, thereby preventing raccoons and other animals access to the bait. 

Two RVNA cutoff levels of ≥0.05 IU/mL (used by CDC [24]) and ≥0.1 IU/mL (suggested by Canadian and European counterparts) were examined. No comparable difference in the results was found when using the higher cutoff (Table 2). For this study, no justifiable reason was found to conclude that using ≥0.05 IU/mL as the cutoff was limiting or accounted for animals with false-elevated RVNA results. There remains much debate about the levels of rabies antibodies that confer resistance to rabies virus infection. No single cutoff level of RVNA is recognized as being invariably protective [31]. Repeated observations that small fractions of animals presenting detectable levels of antibody prior to challenge have shown the animals can still succumb to rabies infection, and conversely that some seronegative animals survive challenge [32,33]. While these discrepancies exist, Blanton et al. [33] did observe that no raccoon succumbed to rabies challenge after vaccination with RABORAL^®^ V-RG, even with an RVNA level of 0.06 IU/mL at the time of challenge. This result indicates to us that a cutoff of ≥0.05 IU/mL for this study was sufficient.

Lower RVNA response in the urban LBSD site may be related to a lower population of raccoons (Figure 3) or a preponderance of private properties surrounded by fences and smaller lots than in the urban HBSD site. To set the bait stations on private property in the LBSD site, the bait stations were placed inside the fences, as requested by property owners. This placement may have reduced the opportunities for raccoons to find the baits. Bozek et al. [34] found raccoons in urban areas had smaller home ranges than those in rural areas. Raccoons in urban habitats have access to anthropogenic food sources and can thereby reduce their foraging distances and patterns. These human food sources may also explain why baits were left in the urban LBSD bait stations. By providing increased bait station density within yards without fences, raccoons likely had easier access to the bait stations in the urban HBSD site, resulting in more baits taken and a significantly higher RVNA response.

Tetracycline biomarker results and RVNA rates were not compared in this study for a few reasons. First, canine teeth and mandibular bone are superior tissues for tetracycline biomarking [20,22], but first premolar teeth were collected for this study as a less intrusive procedure, so that raccoons could be released after full recovery from sedation. Canine tooth sampling would have required euthanasia and eliminated the opportunity to obtain valuable biologic information in future field trials through recaptures. First premolar teeth continue to be the most acceptable, least intrusive sample to collect from live-trapped raccoons. Second, while not noted earlier in this study, unpunctured sachet packets were found at every bait station. The FMP coating was missing, presumably eaten by a raccoon or opossum. This would result in a positive biomarker in the tooth, but no positive RVNA response. Third, background sources could have contributed to tetracycline biomarking. The most likely background tetracycline sources may have included consumption of medicated feeds sometimes used for cattle production and nonspecific fluorescence that may be found naturally [35]. While all study sites had lower biomarker percentages than elevated RVNA percentages, the 2011 urban LBSD site had a higher percentage of biomarkers present than percentage of elevated RVNA. It is unsure why this could be, unless tetracycline is present in the environment or the raccoons were avoiding the vaccine sachet and strictly eating the FMP coating. The sites with higher percentages of elevated RVNA than percentage of tetracycline biomarker may suggest a natural response to rabies in the area, poor tetracycline uptake in the first premolar tooth samples, or, as could be the case in 2012, trapping ‘missed’ animals from 2011 that ingested the vaccine. Additionally, the easier locating of bait stations by the animals, as evidenced by the increased number of baits removed from the bait stations in 2012, could have resulted in the higher percentages of elevated RVNA (Table 1).

The findings from this study support a higher bait station distribution density, to provide greater access to raccoons in urban settings to achieve higher RVNA to meet raccoon rabies management goals. However, additional well-designed studies are required to better understand optimized bait station density and distribution to achieve raccoon rabies elimination in the urban environments that form the mosaic of landscapes on which raccoon rabies occurs.

## Figures and Tables

**Table 1:** Bait station study area designs and results for 2011 and 2012 in rural and urban sites in Florida.

	Rural				Urban			
	2011		2012		2011		2012	
Site (n bait stations; baits/station)	LBSD (9; 75)	HBSD (27; 25)	LBSD (9; 75)	HBSD (27; 25)	LBSD (9; 75)	HBSD (27; 25)	LBSD (9; 75)	HBSD (27; 25)
Baits removed from bait stations (percent of total baits)	513 (76.0%)	620 (91.9%)	675 (100%)	650 (96.3%)	179 (26.5%)	501 (74.2%)	265 (39.3%)	639 (94.7%)
Raccoons trapped	37	37	27	26	32	31	16	31
Percent raccoons RVNA^a^ (*n* positive raccoons)	21.6 (8)	35.1 (13)	44.4 (12)	53.8 (14)	12.5 (4)	45.2 (14)	6.3 (1)	51.6 (16)
Percent raccoons RVNA^b^ (*n* positive raccoons)	21.6 (8)	35.1 (13)	44.4 (12)	53.8 (14)	9.4 (3)	38.7 (12)	6.3 (1)	51.6 (16)
Percent biomarker present (*n* 1st premolar teeth; biomarker present)	14.7 (34; 5)	28.1 (32; 9)	26.9 (26; 7)	30.4 (23; 7)	19.4 (31; 6)	19.2 (26; 5)	0.0 (16; 0)	33.3 (24; 8)

^a^ cut-off of ≥0.05 IU/mL was used to indicate a positive RVNA; ^b^ cut-off of ≥0.1 IU/mL was used to indicate a positive RVNA.

**Table 2:** Comparison of raccoon RVNA (at 75 baits/km^2^) with deployment of LBSD versus HBSD in rural and urban environments in Florida, 2011–2012.

County/Year	Percent RVNA (*n*)	Fisher’s Exact Test
**A**. Showing results using RVNA cutoff of ≥0.05 IU/mL		
Rural 2011 (LBSD vs. HBSD)	21.6 (37) vs. 35.1 (37)	*p* = 0.3024
Rural 2012 (LBSD vs. HBSD)	44.4 (27) vs. 53.8 (26)	*p* = 0.5867
Urban 2011 (LBSD vs. HBSD)	12.5 (32) vs. 45.2 (31)	*p* = 0.0054
Urban 2012 (LBSD vs. HBSD)	6.3 (16) vs. 51.6 (31)	*p* = 0.0031
**B**. Showing results using RVNA cutoff of ≥0.1 IU/mL		
Rural 2011 (LBSD vs. HBSD)	21.6 (37) vs. 35.1 (37)	*p* = 0.3024
Rural 2012 (LBSD vs. HBSD)	44.4 (27) vs. 53.8 (26)	*p* = 0.5867
Urban 2011 (LBSD vs. HBSD)	9.4 (32) vs. 38.7 (31)	*p* = 0.0081
Urban 2012 (LBSD vs. HBSD)	6.3 (16) vs. 51.6 (31)	*p* = 0.0031

**Table 3:** Comparison of raccoon tetracycline deposition (at 75 baits/km^2^) with deployment of LBSD versus HBSD in rural and urban environments in Florida, 2011–2012.

County/Year	Percent Tetracycline-Positive (*n*)	Fisher’s Exact Test Result
Rural 2011 (LBSD vs. HBSD)	14.7 (34) vs. 28.1 (32)	*p* = 0.2344
Rural 2012 (LBSD vs. HBSD)	26.9 (26) vs. 30.4 (23)	*p* = 1.000
Urban 2011 (LBSD vs. HBSD)	19.4 (31) vs. 19.2 (26)	*p* = 1.000
Urban 2012 (LBSD vs. HBSD)	0 (16) vs. 33.3 (24)	*p* = 0.0133
